# Challenges in Founding and Developing Medical School Student-Run Asylum Clinics

**DOI:** 10.1007/s10903-020-01106-2

**Published:** 2020-10-21

**Authors:** Fangning Gu, Emily Chu, Andrew Milewski, Sophia Taleghani, Mehar Maju, Randall Kuhn, Adam Richards, Eleanor Emery

**Affiliations:** 1 David Geffen School of Medicine, 10833 Le Conte Ave, Los Angeles, CA 90095 USA; 2 Weill Cornell Medicine, New York, NY USA; 3 Department of Community Health Sciences, UCLA Fielding School of Public Health, Los Angeles, CA USA; 4 Community Partners International, Research, San Francisco, CA USA; 5 Center for Health Equity Education and Advocacy, Cambridge Health Alliance, Cambridge, MA USA; 6 Department of Internal Medicine, Northern Navajo Medical Center, Shiprock, NM USA

**Keywords:** Asylum seekers, Forensic medical evaluation, Human rights education, Student-run asylum clinics

## Abstract

In response to the rapidly rising number of asylum applications, student-run asylum clinics (SRACs) designed to provide *pro bono* forensic medical evaluations have emerged at medical schools across the United States. Distinct from traditional student-run clinics in the services they provide and in their operational models, SRACs face a unique set of challenges. This study aims to identify the common challenges in building SRACs and to collect insights to inform a structured approach to collaborative problem-solving. This study gathered data from online surveys and semi-structured phone interviews with representative medical student SRAC leaders. 14 clinics participated in the 2017 online survey, 15 clinics in the 2018 online survey, and eight clinics in the 2018–2019 phone interviews. We identified common challenges in five areas: volunteer recruitment, clinic operations, case demand, institutional support, and leadership. SRACs stand to benefit from ongoing extramural collaborations to overcome shared challenges.

**Electronic supplementary material:**

The online version of this article (10.1007/s10903-020-01106-2) contains supplementary material, which is available to authorized users.

## Introduction

Every year, a growing number of individuals flee persecution in their home countries and seek asylum in the United States: The backlog of pending asylum cases grew from 6000 in 2009 to more than 320,000 by the end of 2018 [1]. Although individuals have the legal right to seek asylum in the U.S., asylum seekers bear the burden of proving past persecution or a well-founded fear of persecutions in their home countries [2]. Trained clinicians can perform forensic medical evaluations (FMEs) to identify psychological and physical sequelae of trauma and document their findings in medical-legal affidavits that provide crucial evidence for corroborating a client’s narrative of torture, abuse, or persecution [3]. According to a 2008 study, 89% of asylum applicants with legal representation and an FME were granted protective status, compared to an asylum grant rate of 37.5% among those—with or without legal representation—who did not receive an FME [4]. Despite their critical importance, numerous barriers limit access to FMEs, including a paucity of trained clinicians, infrequent training opportunities, and an inability to pay for forensic evaluations [5].

To address these barriers, by 2020, students at more than 20 medical schools across the country have established Student-Run Asylum Clinics (SRACs) that provide *pro bono* FMEs for asylum seekers [6]. With the support of faculty and Physicians for Human Rights (PHR), these student-led initiatives train clinicians and students to perform FMEs, support asylum-related advocacy and research, and may administer care-referral programs that refer asylum seekers for ongoing healthcare and social services [6]. Although previous articles have discussed the founding and operation of SRACs, a detailed analysis of the challenges faced by SRACs has not yet been undertaken [6–9]. We hypothesized that many SRACs experience a common set of challenges. Through surveys and interviews with student leaders at SRACs across the U.S., this study aims to delineate the challenges encountered by SRACs in founding and growing their clinics and to document best practices for addressing those challenges.

## Methods

The PHR Student Advisory Board (SAB) administered anonymous online surveys to 16 SRACs in 2017 and 18 SRACs in 2018, accounting for all of the established SRACs in the U.S. at those times. Student clinic leaders were invited to provide free-text responses to the question: “Has your clinic faced any challenges during the past year? If so, was your organization able to address those challenges?” 15 and 14 SRACs responded in 2017 and 2018 respectively [6]. We then used the results of these surveys to develop a targeted questionnaire in the form of semi-structured, qualitative phone interviews to explore the context surrounding the self-reported challenges and to pool together tried solutions to those challenges. Among all of the established SRACs, we selected 12 SRACs that represent diverse geographic locations, stages of development, and types of institutions. Of the 12 clinics we contacted, eight responded and participated in the phone interviews in 2019 (“Appendix Table S1”). The interviews included 23 open-ended questions and pre-scripted questions. Many of the interview questions explored themes identified by the PHR SAB surveys. For example, several SRACs indicated difficulties with leadership transitions in the surveys. The phone interviews therefore included open-ended questions asking for a descriptions of the SRAC’s leadership structure, the structure’s evolution, and challenges pertaining to leadership, as well as targeted questions such as “what was your clinic’s experience with leadership transitions?” and “what have you found helpful in facilitating leadership transitions?” The Institutional Review Board at the University of California was consulted and concluded that the data obtained by this study did not constitute human subjects research. The Institutional Review Board at Yale University was consulted and concluded the same for the PHR SAB survey data [6].

We applied thematic, qualitative analysis to the combined results of the PHR SAB surveys and the phone interviews. Two team members (F.G., E.C.) independently extracted challenges from the PHR SAB surveys and the transcripts of the phone interviews. They coded each challenge and constructed a codebook of recurring themes. Codes such as “case scheduling” and “case-referral programs” were grouped into “clinic operation.” Two coders found high similarities in their respective results. They then discussed any discrepancies in coding to achieve consensus. Thematic saturation was reached at around the sixth phone interview, at which point neither coder noted further changes to their codebooks with subsequent interviews. No qualitative analysis software was used. All statistics in this study were calculated in Microsoft Excel (version 14.0.0).

## Results

**Fig. 1:**
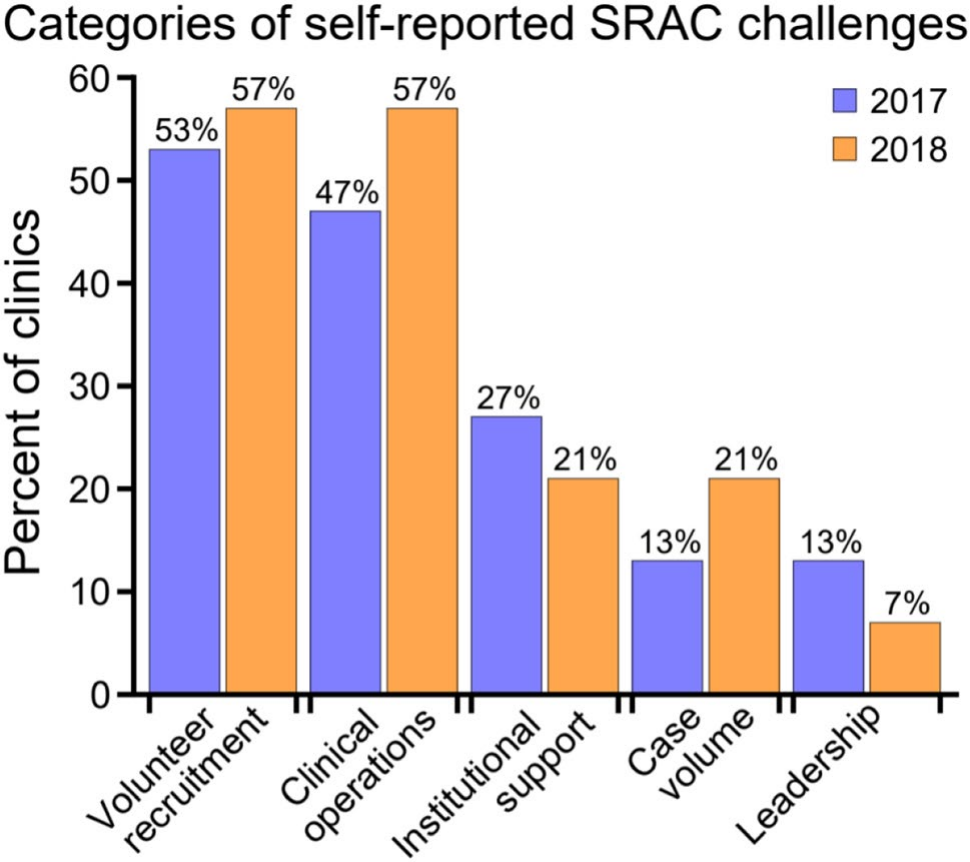
Five themes characterize the challenges reported by Student-Run Asylum Clinics (SRACs) in response to the PHR SAB survey question: “Has your clinic faced any challenges during the past year?” The reported frequencies are calculated as the number of clinics whose responses fell within a given challenge category divided by the number of SRACs that responded to the survey question: 15 out of the 16 (94%) SRACs surveyed in 2017 answered the question and 14 out of 18 (78%) responded in 2018

The common challenges identified across the PHR SAB surveys were grouped into five categories (Fig. 1). Fifteen out of the 16 (94%) SRACs surveyed in 2017 answered the question, and 14 out of 18 (78%) responded in 2018. Eight out of the 12 (67%) contacted SRACs partook in the phone interviews. Table 1 contains additional details and summarizes solutions reported by SRACs to address each challenge.

**Table 1:** Summary of the major challenges faced by SRACs and example solutions

	Specific challenges identified	Solutions used by SRACs
Volunteer Recruitment	Increasing volume of FME requests^a^	Expand recruitment initiatives^b^
	Length of required training	Offer remote-training options
	Needing more clinicians who are comfortable performing mental health evaluation	Train non-mental health providers to perform psychological FMEs
	Retaining clinicians^c^	Engage newly trained clinicians in seminars, continued learning, and social events
	Onboarding process for the newly trained clinicians^d^	Engage newly trained clinicians in mentorship or advocacy while they await shadowing opportunities
Clinical Operations	Time-consuming process for scheduling FMEs	Optimize the scheduling process through technological innovations^e^ Let lawyers and clinicians communicate directly to schedule FMEs
	Initiating care-referral programs that connect clients to resources in their communities	Dedicate student leaders to the task Partner with organizations for referral services
Case Volume	Acquiring cases for newly established clinics^f^	Obtain referrals from PHR and nearby SRACs Balance caseloads across SRACs by centralizing the scheduling process for nearby clinics
	Fluctuating monthly case requests Adjusting to changing immigration policies	Establish relationships with local referral sources^g^
	Increasing numbers of requests to evaluate clients at detention centers	Consider performing FMEs remotely Establish protocols with detention centers
Institutional Support	Concerns that “asylum” in the SRAC’s name will be perceived as political	Adopt a neutral name^h^
	Misconceptions that SRACs provide direct medical care	Clarify that FMEs do not provide direct medical care Avoid using “clinic” in the organization’s name
	Prohibitions of certain types of evaluations conducted on campus	Conduct evaluations off-campus^i^
Leadership	Time commitment for SRAC leaders	Expand the leadership team
	Transitions for SRAC leadership from one class of medical students to the next	Extensively document the clinic’s operations and mentor new leaders^j^ Secure protected time for clinic advisers

^a^One established clinic reported scheduling more than 18 cases/month

^b^Non-MD clinicians can include LCSWs, PAs, NPs, DOs, PsyDs, and PhD-level psychologists

^c^Only 10–30% of trained clinicians subsequently volunteer for a case

^d^Many SRACs required newly trained clinicians to shadow one to three evaluations before independently conducting FMEs, which is often difficult to schedule and lengthens the time to independence

^e^For example, one clinic programmed excel algorithms to streamline case scheduling

^f^SRACs receive one to nine cases per month; newer clinics generally receive fewer cases

^g^Local referrals include legal organizations, law schools, and other asylum clinics

^h^A commonly adopted name is “Human Rights Collaborative”

^i^One commonly used off-campus option is the office of the client’s attorney

^j^Many SRACs allow 6 months to 1 year for leadership transitions

### Volunteer Recruitment

Recruiting enough clinician and medical student volunteers to meet the demands for FMEs—especially psychological FMEs—was the most commonly identified challenge. Many clinics echoed the claim: “[the] number of evaluation requests skyrocketed” between 2017 and 2018, thereby intensifying the pressure to recruit.

The time commitment (5–9 h) to attend the required FME training session hampered recruitment efforts. Despite significant interest, several clinics reported that only 50–70% of the registered participants attended the training. Clinics addressed provider shortages by recruiting peers of active evaluators, non-MD clinicians, and clinicians not affiliated with the institution housing the SRAC.

Another challenge was the lack of trained clinicians who felt comfortable conducting psychological evaluations, which account for approximately half of the FME requests received by SRACs [6]. Many SRACs echoed the claim: “we do not have enough psychiatry volunteers!” Clinics responded by organizing specialized training to help non-mental health providers, including internists, feel more comfortable conducting psychological evaluations.

### Clinical Operations

Developing efficient processes for scheduling FMEs was the second-most frequently identified challenge. Scheduling FMEs constitutes the core operational activity for most clinics, and scheduling procedures vary widely across clinics. The eight clinics that we interviewed by phone reported requiring 2 h on average to schedule each case, with a range of 30 min to 10 h. Many SRACs streamlined their scheduling procedures after receiving helpful workflow tips, such as email templates, from peer clinics.

### Institutional Support

Obtaining institutional support for establishing an SRAC was sometimes fraught with liability concerns and branding restrictions. Partnering with the school’s administration is often crucial for securing funding and on-campus space to conduct FMEs. Many clinics at both public and private universities established a Memorandum of Understanding with the school’s administration and PHR. Two clinics described school deans as “champions” who were crucial for “cutting through red tape.” A number of other clinics, however, reported that their school’s administration raised concerns related to the misconception that SRACs provide direct medical care, which raised questions about liability and the need for medical malpractice insurance. Another clinic reported concerns around the word “asylum,” as their administration was “concerned with legal liability and with [the clinic] being perceived as a political group.” Multiple SRACs found that clarifying their objectives—explaining that FMEs do not provide direct medical care and that medical liability therefore does not apply—and adopting a more neutral name, such as “XYZ Human Rights Collaborative,” was essential for obtaining approval from the school’s administration.

### Case Volume

SRACs were also challenged by fluctuating numbers of FME requests and by tighter deadlines for completing the evaluations, two variables that were directly influenced by changing immigration policies. As one clinic reported: “The changes under Trump make a student-run clinic more challenging than in the past—new cases have a more rapid turnaround time”. Many clinics also reported increasing numbers of FME requests for detained clients, which often have short deadlines and require significant travel time. Many clinics responded by establishing protocols for conducting evaluations at detention centers. Some clinics are exploring ways to conduct FMEs remotely.

### Leadership

Medical student leaders also had difficulties accommodating the significant time commitment demanded by SRACs, particularly during a clinic’s early development. As one clinic leader explained: “It is like a part-time job on top of medical school.” Student leaders committed 10 to more than 20 h per week to their roles. To pursue research, advocacy, and care-referral objectives, some clinics expanded their leadership team.

Many clinics noted the importance of faculty advisors in supporting student leaders. The advisors’ involvement ranged from “reading every email” to being more hands-off. Only one of the interviewed clinics reported that protected time was secured for its faculty advisor.

## Discussion

The number of SRACs in the U.S. has grown considerably in the past decade [6]. SRACs are distinct from other student-run organizations and face a unique set of challenges. Our surveys and interviews identified five major categories of common SRAC challenges: volunteer recruitment, clinical operations, case volume, institutional support, and leadership.

In this paper, we present the first detailed analysis of the challenges that are commonly faced by SRACs across the nation. This analysis can inform clinics of the barriers they might face in each stage of development. Many challenges can be addressed by sharing best practices between SRACs. For example, sharing email templates has helped new clinics streamline their scheduling procedures. Nascent clinics would also benefit from guidance around naming the organization and from access to project proposals containing specific language to dispel the misconceptions and concerns that are frequently voiced by school administrators. To address challenges in recruiting volunteers and fluctuating case demand, SRACs serving the same community could consolidate their scheduling mechanisms and share referrals, volunteer networks, and training resources. The PHR SAB or another centralized organization could facilitate an exchange of best practices and provide updated guidance based on annual surveys.

This study is limited by its small sample size, which precluded stratification of the challenges by a clinic’s geographic location or founding year. By excluding institutions that may have tried but failed to establish SRACs, this study cannot identify barriers that might be insurmountable in certain contexts. Furthermore, clinics that responded to the surveys might be inherently more connected with the rest of the clinic network, which could introduce selection bias in the types of challenges identified. Because the eight interviewed clinics reside on the West (25%) or East (75%) coasts in cities whose asylum grant rates exceed the average national rate [10], this study could suffer from a geographic bias that misses challenges and solutions unique to states with unfavorable grant rates. Because the data were collected from 2017–2019, this study does not address the challenges arising for SRACs from the severe acute respiratory syndrome coronavirus 2 (SARS-CoV-2) pandemic.

As SRACs assume a more substantial role addressing the growing demands for FMEs and providing invaluable education to medical students, they stand to benefit from ongoing extramural collaborations to overcome shared challenges.

## Electronic supplementary material

Below is the link to the electronic supplementary material.

Supplementary file1 (DOCX 16 kb)

## Data Availability

The datasets generated during and/or analyzed during the current study are available from the corresponding author on reasonable request.
