# Supplementary material for: Challenges in Founding and Developing Medical School Student-Run Asylum Clinics
Source: J Immigr Minor Health. 2020 Oct 21;23(1):179–83. doi: 10.1007/s10903-020-01106-2 (PMC7576100; doi:10.1007/s10903-020-01106-2)
Supplement: Supplementary file 1 — Supplementary file1 (DOCX 16 kb) [file 10903_2020_1106_MOESM1_ESM.docx]

**APPENDIX**

| **Table S1:** Demographic information on the eight clinics that participated in the phone interviews | | |
| --- | --- | --- |
| Year Founded | Coast | University |
| 2009-2012 | East | Private |
| 2009-2012 | East | Private |
| 2013-2016 | East | Private |
| 2013-2016 | East | Private |
| 2013-2016 | East | Private |
| 2017-2020 | West | Private |
| 2017-2020 | West | Public |
| 2017-2020 | East | Private |

* Year founded is given in ranges to prevent identification of any specific clinic
